# Higher risk of ACL rupture in amateur football compared to professional football: 5-year results of the ‘Anterior cruciate ligament-registry in German football’

**DOI:** 10.1007/s00167-021-06737-y

**Published:** 2021-09-15

**Authors:** Dominik Szymski, Leonard Achenbach, Johannes Zellner, Johannes Weber, Matthias Koch, Florian Zeman, Gunnar Huppertz, Christian Pfeifer, Volker Alt, Werner Krutsch

**Affiliations:** 1 Department of Trauma Surgery, University Medical Centre Regensburg, Franz-Josef-Strauss-Allee 11, 93053 Regensburg, Germany; 2 Department of Orthopedics, König-Ludwig-Haus, Julius-Maximilians-University Würzburg, Würzburg, Germany; 3 Department of Trauma Surgery, Caritas Hospital St. Josef, Regensburg, Germany; 4 Centre for Clinical Studies, University Hospital Regensburg, Regensburg, Germany; 5 SportDocsFranken, Nürnberg, Germany

**Keywords:** Anterior cruciate ligament, Knee injury, Injury prevention, Football

## Abstract

**Purpose:**

Anterior cruciate ligament (ACL) injuries are a common severe type of football injury at all levels of play. A football-specific ACL registry providing both prospective ACL injury data according to the skill level and risk factors for ACL injury is lacking in the literature.

**Methods:**

This study is based on the prospective ‘ACL registry in German Football’ implemented in the 2014–15 season. Professional (1st–3rd league), semi-professional (4th–6th league) and amateur leagues (7th league) were analysed regarding the incidence and risk factors for ACL injuries. Injuries were registered according to the direct reports of the injured players to the study office and double-checked via media analysis. After injury registration, the players received a standardised questionnaire. Data were analysed from the 2014–15 to the 2018–19 football season.

**Results:**

Overall, 958 ACL injuries were registered during the 5-year study period. The incidence of ACL injuries was highest in amateur football (0.074/1000 h football exposure) compared to professional (0.058/1000 h; *p* < 0.0001) and semi-professional football (0.043/1000 h; *p* < 0.0001). At all skill levels, match incidence (professional: 0.343; semi-professional: 0.249; amateur: 0.319) was significantly higher than training incidence (professional: 0.015; semi-professional: 0.004; amateur: 0.005). Major risk factors were previous ACL injury (mean: 23.3%), other knee injuries (mean: 19.3%) and move to a higher league (mean: 24.2%).

**Conclusion:**

This sports-specific ACL registry provides detailed information on the incidence and risk factors for ACL injuries in football over five years. Risk factors are skill level, match exposure, move to a higher league and previous knee injury. These factors offer potential starting points for screening at-risk players and applying targeted prevention.

**Level of evidence:**

II.

## Introduction

Anterior cruciate ligament (ACL) ligament injuries are one of the most common types of football injuries at all levels of play and associated with severe mid-term and long-term effects. Injury severity in football can be defined by absence from football for 7–12 months [18, 20, 23] or by the mid-term effects of injuries, for instance a high rate of performance reduction [18, 34]. In addition, the long-term effects include a high rate of gonarthrosis in the affected players [36]. Knowledge on ACL injuries is still rather limited despite the large number of scientific research reports available; thus, the incidence of ACL injuries in football remains high and is still associated with long periods of absence from playing.

General knowledge on the mechanisms of ACL injuries is well known [3, 24], but detailed information at the different football levels is still lacking but would be necessary for implementing specific injury prevention measures [24]. Epidemiological injury registrations represent an important data source to obtain sports-specific and injury-related information on the mechanisms and risk factors of specific injuries. Various ACL registries were established in the Scandinavian countries, Luxemburg, the United Kingdom, New Zealand, but also in the United States [5, 8, 25, 26, 28] and provide detailed knowledge about the prevalence, basic epidemiology as well as diagnostic and surgical issues of the general population affected by ACL injuries. Although the ACL registries established in the above-mentioned countries also include some sports-specific sub-analyses for specific types of sports such as football, they do not provide any prospective longitudinal data on ACL injuries sustained by a specific population such as football players, in particular according to the different football levels, which represents a huge gap of knowledge in this scientific field. A prospective longitudinal cohort study by the UEFA (UEFA Injury Study) on the population of professional football players included seasonal investigations into the epidemiology of ACL injuries [7, 33, 35] but did not provide any data on the skill levels below the UEFA Champions League. Insight into the risk factors for ACL injury has been provided by several studies that included prospective [19] or retrospective [21] data on ACL injuries over one or more seasons. The general introduction of preventive measures into training in all performance classes, critical questioning of previous measures and continuous injury monitoring are particularly relevant. Yet, in amateur and recreational football as the foundation of football with hundreds of millions of players worldwide, scientific registration of ACL injuries is still lacking completely [19, 34].

Therefore, a national football-specific ACL registry was established for professional and amateur football players in Germany in the season 2014–15. The aims of this registry were to enable the prospective and longitudinal registration of ACL injuries in national football and to analyse football-specific epidemiological data on injury incidence and mechanisms as well as risk factors for ACL injury at different levels of play.

## Materials and methods

**Fig. 1:**
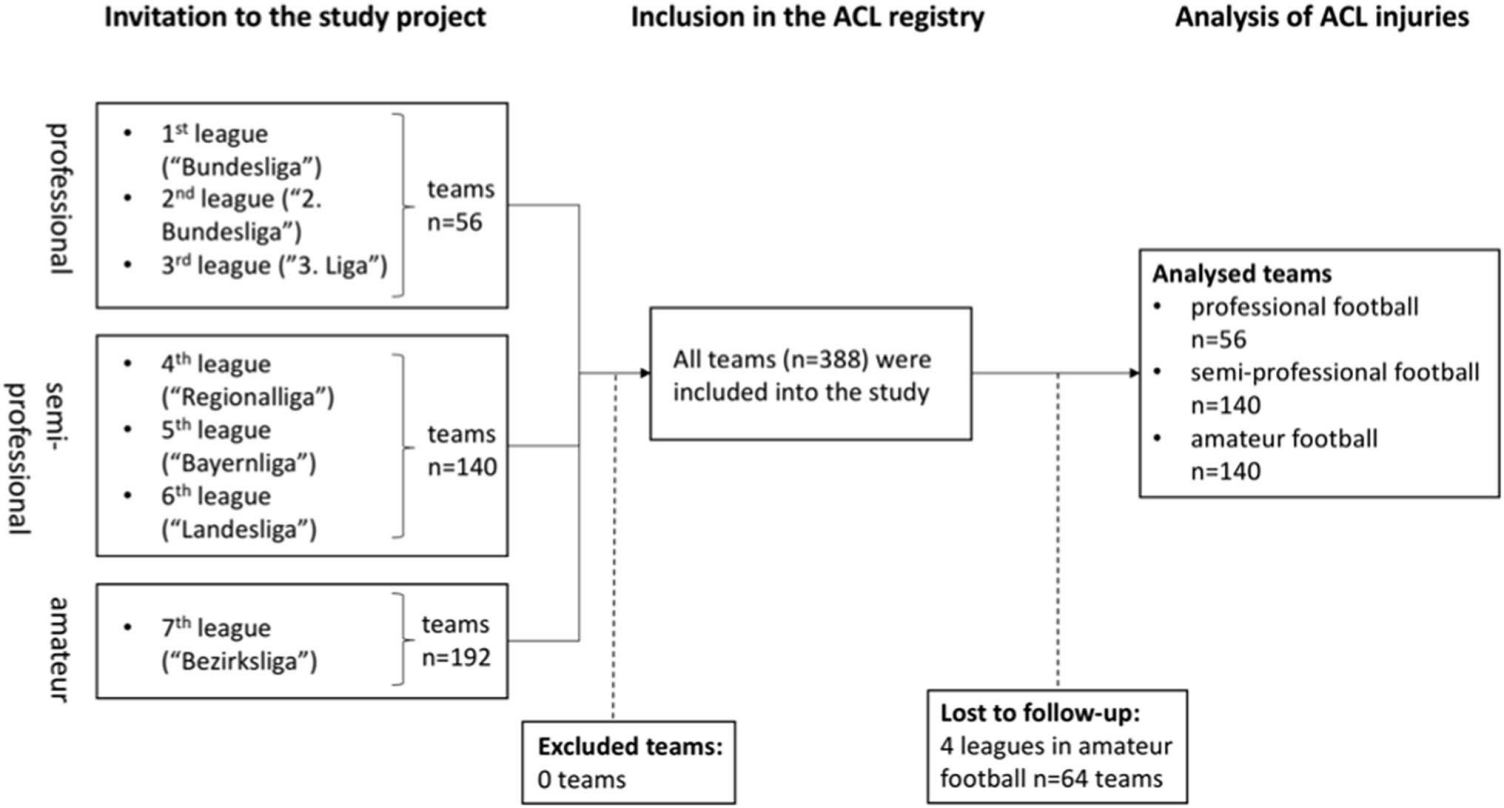
Overview of invitation, inclusion and analysis of the study population of the German registry of anterior cruciate ligament injuries (2014–2019) according to the different football levels

This prospective cohort study is based on the nationwide ACL registry in German football (‘Kreuzbandregister im Deutschen Fußball’) launched at the beginning of the 2014–15 season [31]. The study design of the National ACL registry in football was approved by the Ethics Committee of the University of Regensburg (ID: 10-37_5-101). All professional football clubs in Germany (1st–3rd league) and all non-professional clubs of the Bavarian Football Association in men’s football were invited to participate in the study and to report any new ACL injuries of their players. From the 2014–15 season onwards, the study included all male football players with a new ACL injury who had actively played in the German men's professional leagues (1st–3rd men's professional league) or in the Bavarian amateur leagues (4th–7th men’s amateur league and below). Players with an ACL injury were excluded from prospective injury registration if they had not played at least one competitive match in one of the above-mentioned leagues. All levels from the 1st to the 6th league and eight of the 12 leagues of the 7th playing level of men’s football were included for further analysis. Four leagues of the 7th playing level were excluded from analysis because of non-responsiveness and thus lack of injury data. The study population was divided into German men’s professional (1st–3rd league), Bavarian semi-professional (4th, and all 5th and 6th leagues) and Bavarian amateur football (7th leagues). Professional football players were defined as full-time athletes with a contract, a salary and full insurance and semi-professional football players as paid players with a contract and insurance but no sufficient income to live on, while amateur players do not receive any financial salary (Fig. 1).

Before the beginning of the study in 2014 and the start of ACL injury registration, all football clubs and their team physicians had received an invitation to participate in the study and information on the aim, design and methods of ACL registration. All participants were annually reminded to take part in the study and to report new injuries to the study office. Additionally, the study design was presented to the team physicians of the professional football clubs at medical conferences on professional football and during educational courses at the semi-professional and amateur football levels [15, 31].

**Fig. 2:**
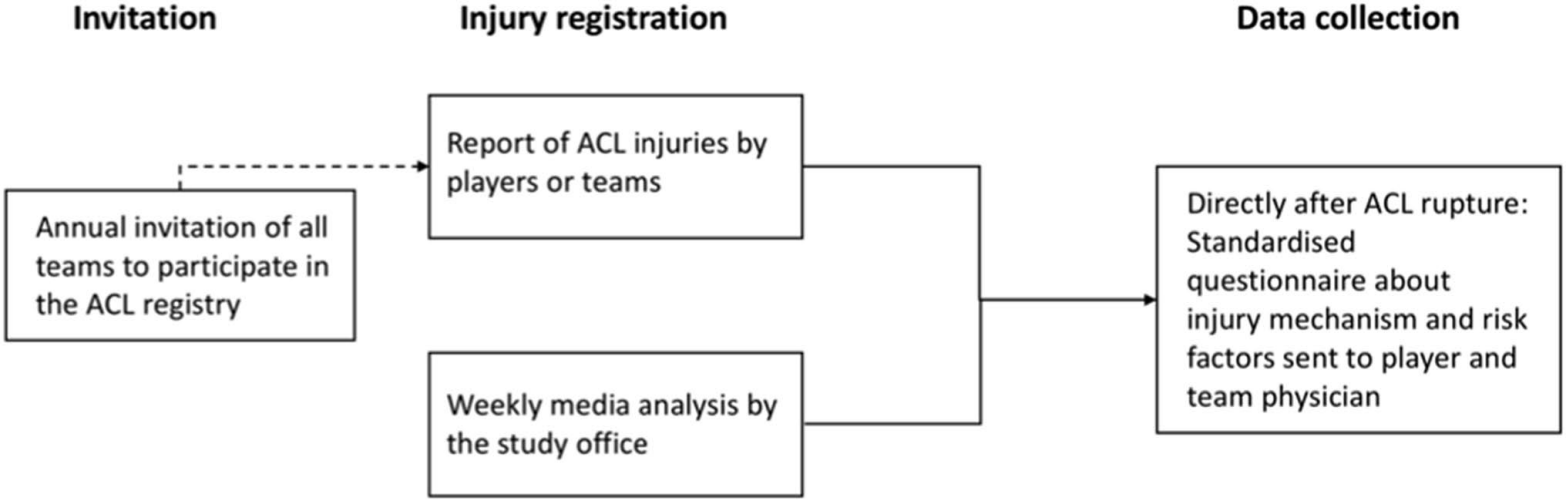
Flow chart of anterior cruciate ligament (ACL) injury registration and data collection in Germany between 2014 and 2019

After the occurrence of an ACL injury, a member of the football club (the team physician, the injured player or a club official) reported the injury to the study office. Because of the size of the study population, the different medical settings of an inhomogeneous study population and the expected varying compliance of different players and clubs to the study guidelines, ACL injuries could not only be registered by players or club members but also by means of national media reports. Each week, national media reports on professional, semi-professional and amateur football were systematically screened in various German online and print media. The use of online and print media for collecting data on severe injuries has been previously described and validated for professional football [2, 14, 18] as well as for other professional team sports [30]. In this ACL registry, such data were only used to double-check the injury reports provided by the clubs. After the registration of a new ACL injury either through direct notification by the players and clubs or according to media reports, a standardised questionnaire [27] in digital or paper form was sent to the injured player or the responsible team member (Fig. 2). The injury reports for this study were adapted according to the commonly used injury report protocol in football established by Fuller et al. (2006) and according to previous epidemiological injury studies of this study group [7, 9, 12–14, 16, 21, 22, 27, 28]. The first protocol, which was sent directly after an injury, included anthropometric and football-specific data, any complaints before the injury, the player’s past medical sports history, external and internal risk factors for ACL injury and detailed information about diagnostics, treatment and the occurrence of the ACL injury. Injury mechanism was categorized into direct, indirect and no-contact injury:

- Direct contact injury: direct application of force from the teammate or opponent or an object to the injured or an adjacent body region
- Indirect contact injury: external force exerted by another person or an object is involved immediately before or during the injury with influence on the athlete's natural course of movement and thus indirectly caused the situation that led to the injury
- No contact injury: if the injury is caused by an event without the application of force by another player or object

Risk factors for ACL injury were divided into physical deficits and short-term changes to address both individual anatomical and physical issues as well as recent changes in exposure and load. All knee injuries with a time loss of at least 2 weeks were classified as prior knee injury. If a club or player failed to provide the required injury data, the respective information was obtained from available media reports.

Injury incidence was calculated per 1000 h of exposure during matches and training as well as the sum of training sessions and matches [9, 11]. Match exposure was calculated by individual analysis of the official match schedules of each football league. No data on the training exposure of individual players or teams were available in this registry. Training exposure was therefore calculated by means of literature reviews and the personal experience of the authors from previous studies of all football levels [1, 12, 14, 19, 21, 22]. Prevalence was defined as the proportion of injuries within the total population. No sample size was calculated because the aim of the study was to recruit as many injured players of the national and regional football associations as possible for the analysed seasons. The study office used the RedCap-System for data management. All analyses for this report were conducted using IBM SPSS Statistics, version 26.0.

## Results

The study population consisted of 46,482 players (mean 9296 per season), 958 ACL injuries were registered in the five consecutive seasons between 2014 and 2019. Four hundred eighty three (50.4%) ACL injuries were registered in recreational amateur football leagues (mean: 60.4 per single league in 5 years), 198 (20.7%) in professional and 277 (28.9%) in semi-professional football leagues. The anthropometric overview of the study population showed comparable physical conditions of the players with ACL injury (Table 1).

**Table 1:** Anthropometric data of German football players with anterior cruciate ligament injury between 2014 and 2019 (*n* = Number of ACL injuries)

	Professional *n* = 198	Semi-professional *n* = 277	Amateur *n* = 483
	*n* ± SD (min; max)	*n* ± SD (min; max)	*n* ± SD (min; max)
Age in years	25.04 ± 4.4 (18.8; 43)	24.48 ± 3.9 (17; 44)	25.31 ± 4.2 (16; 53)
Weight in kg	78.36 ± 7.4 (65; 92)	75.71 ± 6.7 (40; 93)	77.63 ± 12.4 (45; 115)
Height in cm	182.1 ± 6.8 (172; 195)	179.7 ± 5.8 (153; 196)	178.6 ± 8.4 (150; 200)
BMI in kg/m^2^	23.45 ± 1.2 (20.52; 25.46)	23.36 ± 1.7 (17.09; 26.88)	24.98 ± 2.0 (17.16; 34.72)
Experience in years	18.69 ± 4.0; (8; 30)	17.72 ± 3.9 (8;30)	17.30 ± 4.5 (1; 45)

In professional football, the highest training exposure (33.5.3 h per player per season) as well as significant higher match exposure (29.3 h; *p* < 0.001) was registered. Injury mechanism differed between all levels of play with majority of no-contact ACL ruptures in amateur players (*n* = 257; 53.2%), indirect contact injuries in semi-professional (*n* = 108; 39.1%) and primarily direct contact lesions in professional football players (*n* = 95; 48.0%) (Table 2).

**Table 2:** Football-specific data and information on therapy of players with anterior cruciate ligament injury in Germany between 2014 and 2019

	Professional *n* = 198	Semi-professional *n* = 277	Amateur *n* = 483
Position	*n* (%)	*n* (%)	*n* (%)
Goalkeeper	12 (6.1)	10 (3.6)	18 (3.7)
Defender	69 (34.8)	80 (28.9)	160 (33.1)
Midfielder	68 (34.3)	115 (41.5)	221 (45.8)
Striker	49 (24.8)	72 (26.0)	84 (17.4)
Mean football exposure of every player	Per player in hours per season	Per player in hours per season	Per player in hours per season
Training	335.3	286.6	201.6
Match	29.3 ± 2.8	20.3 ± 3.5	20.4 ± 0
Surgical Treatment *n* (%)	195 (98.5)	250 (90.3)	446 (92.3)
Injury mechanism	*n* (%)	*n* (%)	*n* (%)
Direct contact	95 (48.0)	58 (20.9)	106 (22.0)
Indirect contact	34 (17.2)	108 (39.1)	106 (22.0)
No contact	61 (30.8)	94 (33.9)	257 (53.2)
Un-rememberable	8 (4.0)	17 (6.1)	14 (2.8)

**Fig. 3:**
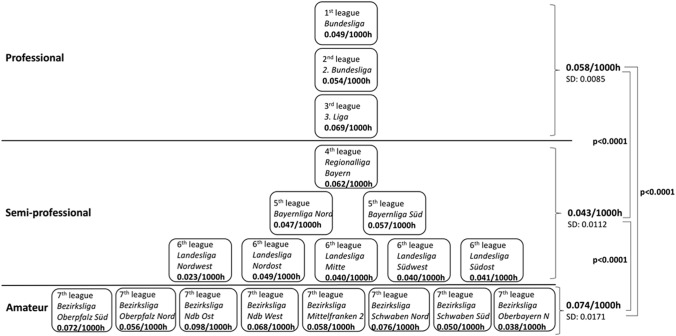
Incidence of anterior cruciate ligament injuries in the different football leagues in Germany between 2014 and 2019 (football exposure in training and matches)

ACL injury incidences not only significantly differed within amateur football leagues (min: 0.038; max: 0.098; *p* < 0.001), but were overall significantly higher than in the professional leagues (0.058/1000 h; *p* < 0.0001). In professional football, the incidence of ACL injuries decreased from the 1st league (0.049) to the 3rd league (0.069); in contrast, the semi-professional football level showed a reverse trend with a high incidence of ACL injuries in the 4th league (0.062/1000 h) and the lowest rates in the 6th league (0.023–0.049; Fig. 3).

**Fig. 4:**
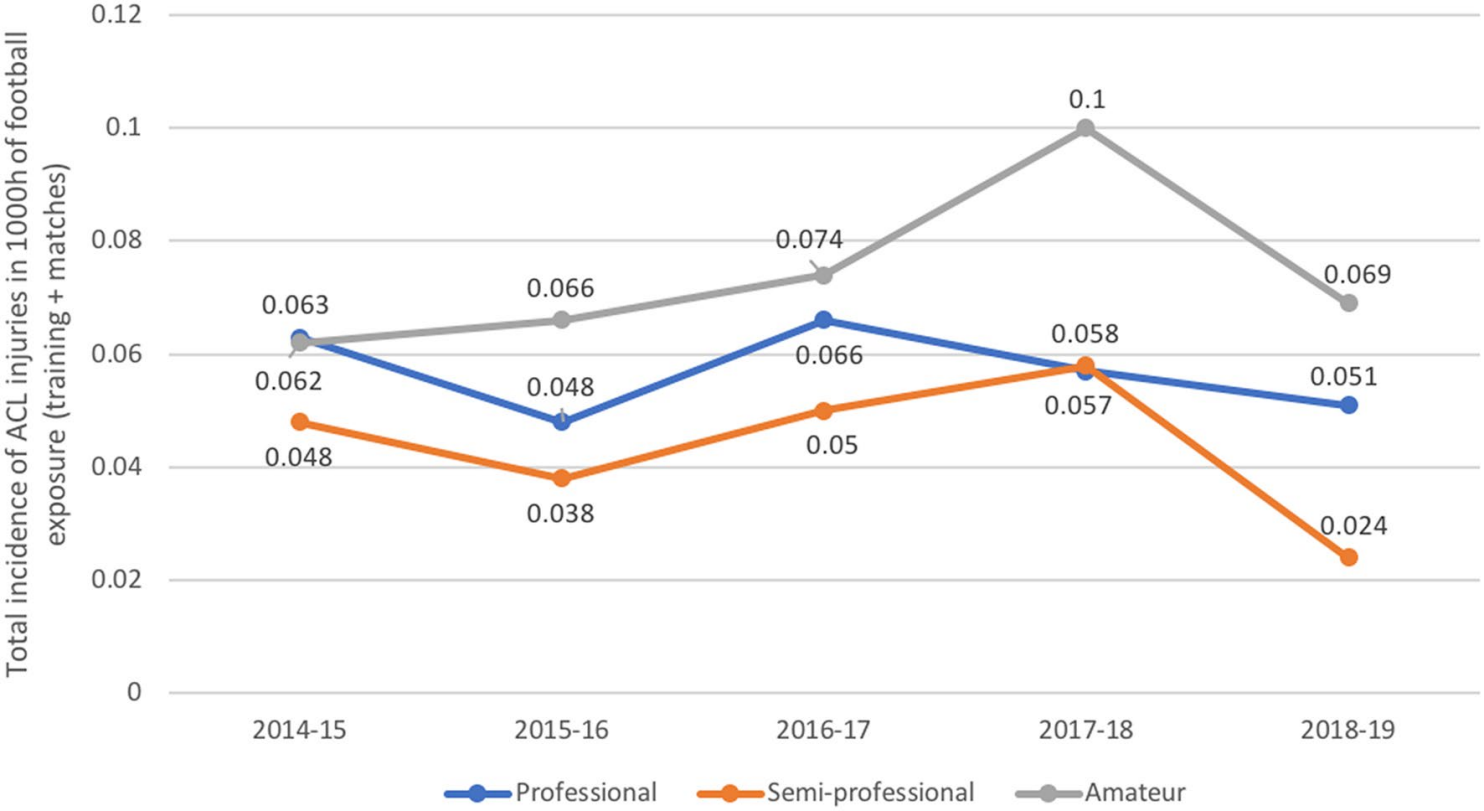
Overall incidence of anterior cruciate ligament injuries at different football levels in Germany between 2014 and 2019

**Fig. 5:**
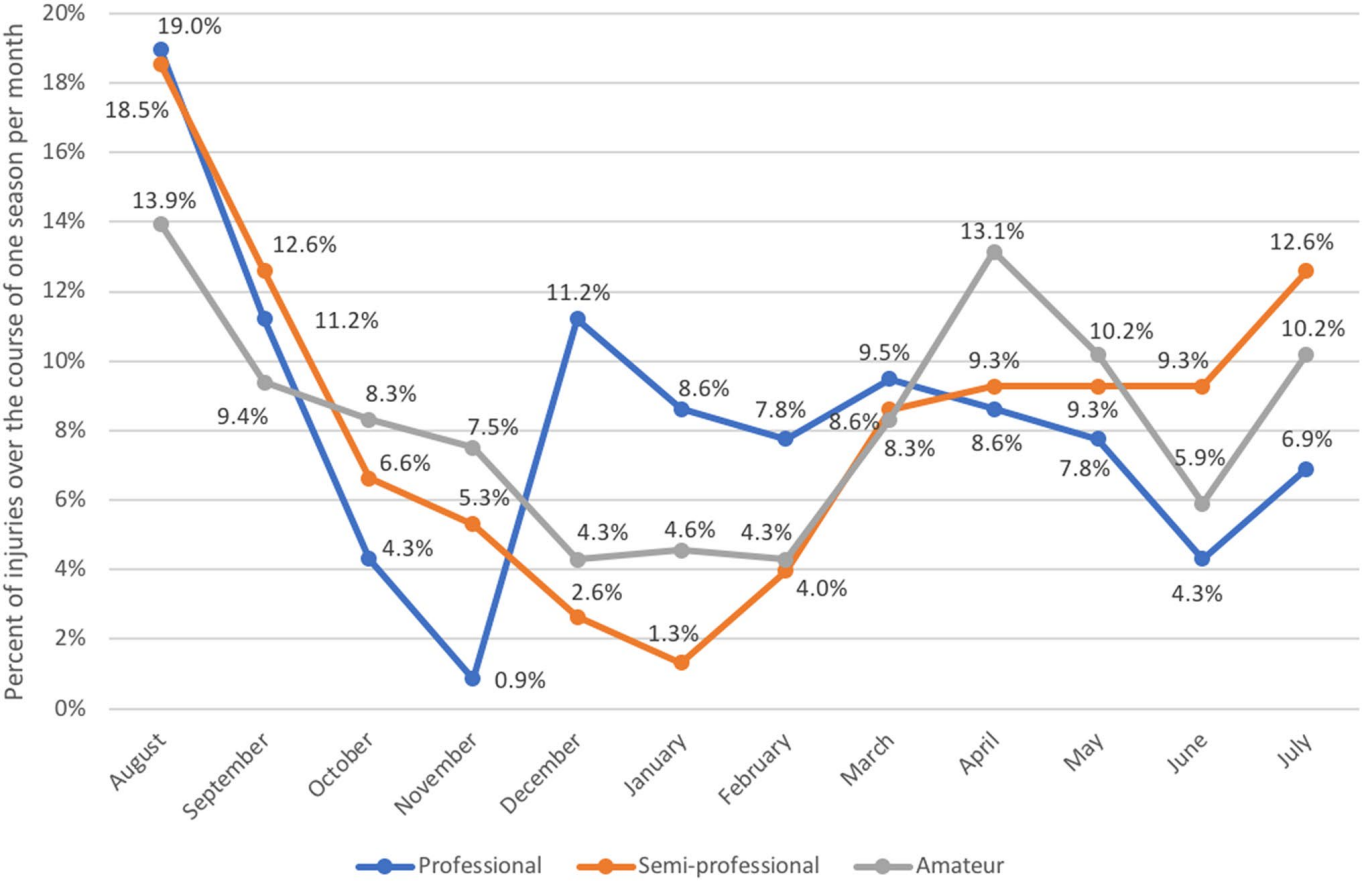
Proportion of ACL injuries for each month over the course of one season (in percent)

Differentiating ACL injuries according to the situation in which they occurred yielded a significant times higher number of injuries in matches than in training sessions (*p* < 0.0001; Table 3). Amateur football leagues showed a slight increase in the incidence of ACL injuries over the time and professional and semi-professional leagues a slight decrease during the study period (Fig. 4). Over the course of a season, highest rates were registered in the beginning of the season in August and September in all levels (professional: 30.2%; semi-professional: 31.1%; amateur: 23.3%) (Fig. 5).

**Table 3:** Incidence of anterior cruciate ligament injuries at the different football levels per 1000 h football exposure in Germany between 2014 and 2019

	2014–15	2015–16	2016–17	2017–18	2018–19	Total
**Professional**						
Match	0.557	0.312	0.111	0.268	0.468	0.343
Training	0.014	0.023	0.006	0.016	0.018	0.015
Overall	0.063	0.048	0.066	0.057	0.051	0.058
**Semi-professional**						
Match	0.388	0.047	0.212	0.271	0.330	0.249
Training	0.005	0.007	0.003	0.004	0.003	0.004
Overall	0.048	0.037	0.049	0.057	0.024	0.043
**Amateur**						
Match	0.622	0.054	0.149	0.406	0.365	0.319
Training	0.005	0.008	0.003	0.007	0.003	0.005
Overall	0.062	0.066	0.074	0.100	0.069	0.074

The most important risk factors for ACL injury in all football leagues were previous ACL injury (mean: 23.3%, SD: 0.99) and other previous knee injuries (mean: 19.3%, SD: 1.72). A further risk factor was absence from football for more than 4 weeks before ACL injury (also scheduled breaks included), which was observed in 20% of players with ACL injury (mean: 22.0%, SD: 1.31). With regard to short-term changes before ACL injury, the move to a higher league involved a higher risk of ACL injury in semi-professional (*n* = 79; 34.8%; OR: 1.55) and professional football (*n* = 54; 27.3%; OR: 1.45) compared to amateur football (*n* = 99; 20.5%; Table 4).

**Table 4:** Risk factors for anterior cruciate ligament injury at different football levels in Germany between 2014 and 2019

	Professional *n* = 198	Semi-Professional *n* = 277	Amateur *n* = 483	Total Football *n* = 958
	*n* (%)	*n* (%)	*n* (%)	*n* (%)
Physical deficits				
Previous ACL injury	47 (23.7)	59 (26.0)	117 (24.2)	223 (23.3)
Other previous knee injury (same side)	45 (22.7)	42 (18.5)	98 (20.3)	185 (19.3)
Absence from football for more than 4 weeks in the 6 months before ACL injury	42 (21.2)	51 (22.5)	118 (24.4)	211 (22.0)
Short-term changes				
Move to a higher league	54 (27.3)	79 (34.8)	99 (20.5)	232 (24.2)
Higher match exposure than usual	47 (23.7)	51 (22.5)	91 (18.8)	189 (19.7)
More than 2 matches in the 7 days before ACL injury	12 (6.1)	34 (15.0)	59 (12.2)	105 (11.0)

## Discussion

The most important findings of this study are the differences in the incidence and risk factors for ACL injuries, which have been analysed with regard to the different football leagues for the first time. As the sport-specific registry with an exclusive population of football players only, this ACL registry represents a novel addition to the already existing ACL registries that include the general population of different countries [5, 8, 26–28]. Information documented in this ACL registry is league-specific injury incidences, sports-specific injury mechanisms and risk factors for ACL injuries, which are less frequently recorded in other registries. In contrast to other time-limited study projects on ACL injuries over one season [16, 19, 22], this ACL registry provides prospective and longitudinal injury data over more than one season, thus representing an adequate basis for trends regarding the occurrence of ACL injuries in all levels of play.

This study provides data on the difference in the incidence of ACL injuries between professional, semi-professional and amateur football in Germany. Amateur football was found to have the highest incidence of ACL injuries and therefore the highest need of injury prevention strategies. The general lack of adequate injury prevention strategies in amateur football may be due to the ignorance of the importance of prevention exercises as well as the lower level of education of the coaching staff and the lower pro-activeness of players at amateur levels a reason for higher rates of incidence in these classes [22].

This study also shows differences in the incidence of ACL injuries between the three professional levels of play, i.e. a trend towards a lower incidence in the 1st and 2nd league than in the 3rd league, which may be a sign for the higher professionalism in implementing prevention strategies in the two highest leagues. Furthermore, the incidences of ACL injuries vary widely between the eight amateur leagues of the 7th playing level and lead to the assumption that they are based on normal deviations. The incidences of ACL injuries of amateur and professional football players found in this study correspond to the incidences at these skill levels described in previous single season-studies [16, 19, 22].

This alarming situation of increasing incidences over 5 years in lower leagues should be addressed by football associations, clubs, coaches as well as players by implementing adequate prevention strategies. The basis of each prevention concept is detailed knowledge about the incidence and mechanism of specific injuries [17, 24]. This study shows the pressing need for implementing strategies for preventing ACL injuries in amateur football. The general trend towards a considerable decrease in the incidence of ACL injuries has not only been found in this study for professional national football but also recently for general injury rates at the UEFA Champions League level [7, 33, 35]. Trends and developments of injury frequencies represent one of the most powerful tools to control and validate previously implemented or current prevention strategies in football.

One of these trends was obtained when observing the rate of ACL injuries over the course of one season. In particular, at the beginning of each season, higher rates of ACL ruptures were found. In semi-professional and amateur leagues, due to climate conditions, a prolonged winter break is scheduled. During this time also, a reduced number of injuries were registered in this registry, while at the start of the second period of the season also, a second peak of ACL ruptures was found. In particular, this part of the season, with restart of match exposure after the season or winter break, has to be watched critically with respect to enhanced prevention.

An increasing percentage of no-contact injuries in lower leagues and classes was registered, while direct contact ACL ruptures were in particularly found in professional football. In literature, majority of injuries were described as no-contact injury with mainly valgus knee loading [3, 24]. The high rate of direct contact injuries in professional football in our study, offers an approach for injury reduction by focus on triggering situations like tackling. However, with the reduction of no-contact injuries in professional football by compensation of neuromuscular and physical deficits [3, 10, 13, 19, 29], recent results of scientific literature seem to be implemented into training in professional athletes. Almost doubling the proportion in amateur footballers with no-contact injuries underlines this point and shows that even in this performance group, a reduction can still be achieved through the evidence-based application of specific training sessions.

Risk factors for football injuries are deficits in physical performance [10, 24, 29] as well as short-term changes in the neuromotorical ability of a player [19] that may influence movement patterns and consecutively increase the risk of injury. This study shows previous knee or ACL injury and insufficient fitness due to absence from football for more than 4 weeks to be markers for physical deficits. The scientific literature includes many epidemiological injury reports about the causes of re-injuries [4, 6, 32], and insufficient fitness [13] has been described as one of them. This fact, which was also seen in nearly 20–25% of our study participants with ACL injuries, has thus been investigated as a risk factor in interventional studies. Short-term changes in neuromotorical ability are a further important risk factor for injuries, especially knee injuries [19]. This study quantified the influence of short-term increases in physical requirements—for instance, after the move to a higher league—with more than 25% and therefore as being relevant for injury prevention. Such increases in physical requirements should be addressed by adequately integrating players into a new club and by giving them sufficient time to adapt to a higher level of play.

This national ACL registry has several benefits but also some limitations. One major limitation is the determination of training exposure using publications published by this study group regarding the same levels of play. The exact registration of training exposure for each of the almost 50,000 players is not possible and therefor a generalization of training exposure for same divisions was obtained. This provides a potential mechanism to skew the results. The data on ACL injuries were collected by means of two different registration methods, such as injury reports provided by the clubs or players and media screening by the study office. Such double-checking measures ensure the highest possible number of correctly identified ACL injuries at each level of play. Thus, when different sources are used to record and confirm ACL injuries, it is likely that injury frequencies are more accurately documented in professional football than at lower skill levels or in women’s football, data that are currently still missing in this registry. Whether we have a strict screening method for the detection of ACL injuries in different playing level, it remains in the end still unclear, if we catch 100% of all ACL injuries or only a percentage. However, with the use of two methods and a double-check of injuries, we tried to detect and confirm as much ACL ruptures as possible. For detailed information about ACL injuries besides their incidence, football players receive an injury protocol that enables the investigation of factors influencing ACL injuries and a treatment protocol to provide information about treatment and the rehabilitation process. Such standardised protocols also show the differences in the compliance of players and clubs at the different skill levels, which is another limitation of this registry. When injury protocols could not be filled in by the injured player because of contact problems, it was possible to obtain a minimum amount of information by means of standardised media analysis (e.g. playing level, match/training injury, previous playing level). This method has been previously scientifically published and validated by Krutsch et al. (2020) for media reports in professional football and can also be applied to other skill levels and types of sports [14, 30]. However, in amateur players, detailed data by analysis of media data are hard to obtain and represent a deficit in the data set, when the player did not fulfil the questionnaire on his own.

In particular, the presentation of the different risk factors in relation to anterior cruciate ligament injuries in footballers of different performance levels shows the clinical relevance of this work. Amateur players thereby showed an increased risk. For this reason, prevention strategies in football should be tailored to the needs of players according to their level of play [16]. The general introduction of preventive measures into training in all performance classes, critical questioning of previous measures and continuous injury monitoring are relevant. Players who already exhibit some of the revealed risk factors for ACL injuries should be monitored more critically and provided with specific prevention and training routines to reduce their risk.

## Conclusion

Incidences of ACL injuries are still high in all football leagues, especially during match situations. This football-specific ACL registry found skill level, match exposition, move to a higher playing level and previous knee injuries to be risk factors for ACL injuries in football. Amateur players with these influencing factors are particularly at risk and should be subjected to targeted prevention. Due to differences in exposure and load as well as risk factors, injury prevention concepts should be tailored to the needs of players according to their level of play. Further prospective investigations with a control group are needed to evaluate the value of each risk factor.
